# Polyzwitterion-grafted decellularized bovine intercostal arteries as new substitutes of small-diameter arteries for vascular regeneration

**DOI:** 10.1093/rb/rbae098

**Published:** 2024-08-22

**Authors:** Yuan Xia, Zilong Rao, Simin Wu, Jiayao Huang, Haiyun Zhou, Hanzhao Li, Hui Zheng, Daxin Guo, Daping Quan, Jing-Song Ou, Ying Bai, Yunqi Liu

**Affiliations:** Division of Cardiac Surgery, Cardiovascular Diseases Institute, The First Affiliated Hospital, Sun Yat-sen University, Guangzhou 510080, China; Guangdong Engineering Technology Research Centre for Functional Biomaterials, Key Laboratory for Polymeric Composite & Functional Materials of Ministry of Education, School of Materials Science and Engineering, Sun Yat-sen University, Guangzhou 510006, China; Guangdong Engineering Technology Research Centre for Functional Biomaterials, Key Laboratory for Polymeric Composite & Functional Materials of Ministry of Education, School of Materials Science and Engineering, Sun Yat-sen University, Guangzhou 510006, China; Department of Medical Ultrasound, The First Affiliated Hospital, Sun Yat-sen University, Guangzhou 510080, China; Department of Cardiac Surgery, The First Affiliated Hospital, Guangzhou Medical University, Guangzhou 510160, China; Department of Cardiac Surgery, The First Affiliated Hospital, Guangzhou Medical University, Guangzhou 510160, China; Department of Cardiac Surgery, The First Affiliated Hospital, Guangzhou Medical University, Guangzhou 510160, China; Department of Cardiac Surgery, The First Affiliated Hospital, Guangzhou Medical University, Guangzhou 510160, China; Guangdong Engineering Technology Research Centre for Functional Biomaterials, Key Laboratory for Polymeric Composite & Functional Materials of Ministry of Education, School of Materials Science and Engineering, Sun Yat-sen University, Guangzhou 510006, China; Division of Cardiac Surgery, Cardiovascular Diseases Institute, The First Affiliated Hospital, Sun Yat-sen University, Guangzhou 510080, China; National-Guangdong Joint Engineering Laboratory for Diagnosis and Treatment of Vascular Diseases, NHC key Laboratory of Assisted Circulation and Vascular Diseases (Sun Yat-sen University), Key Laboratory of Assisted Circulation and Vascular Diseases, Chinese Academy of Medical Sciences, Guangdong Engineering Technology Centre for Diagnosis and Treatment of Vascular Diseases, Guangzhou 510080, China; Guangdong Provincial Key Laboratory of Brain Function and Disease, Zhongshan School of Medicine, Sun Yat-sen University, Guangzhou 510080, China; Guangdong Engineering Technology Research Centre for Functional Biomaterials, Key Laboratory for Polymeric Composite & Functional Materials of Ministry of Education, School of Materials Science and Engineering, Sun Yat-sen University, Guangzhou 510006, China; Department of Cardiac Surgery, The First Affiliated Hospital, Guangzhou Medical University, Guangzhou 510160, China

**Keywords:** small-diameter vascular graft, decellularization, intercostal artery, surface modification, PMPC

## Abstract

Coronary artery bypass grafting is acknowledged as a major clinical approach for treatment of severe coronary artery atherosclerotic heart disease. This procedure typically requires autologous small-diameter vascular grafts. However, the limited availability of the donor vessels and associated trauma during tissue harvest underscore the necessity for artificial arterial alternatives. Herein, decellularized bovine intercostal arteries were successfully fabricated with lengths ranging from 15 to 30 cm, which also closely match the inner diameters of human coronary arteries. These decellularized arterial grafts exhibited great promise following poly(2-methacryloyloxyethyl phosphorylcholine) (PMPC) grafting from the inner surface. Such surface modification endowed the decellularized arteries with superior mechanical strength, enhanced anticoagulant properties and improved biocompatibility, compared to the decellularized bovine intercostal arteries alone, or even those decellularized grafts modified with both heparin and vascular endothelial growth factor. After replacement of the carotid arteries in rabbits, all surface-modified vascular grafts have shown good patency within 30 days post-implantation. Notably, strong signal was observed after α-SMA immunofluorescence staining on the PMPC-grafted vessels, indicating significant potential for regenerating the vascular smooth muscle layer and thereby restoring full structures of the artery. Consequently, the decellularized bovine intercostal arteries surface modified by PMPC can emerge as a potent candidate for small-diameter artificial blood vessels, and have shown great promise to serve as viable substitutes of arterial autografts.

## Graphical Abstract

**Figure:**
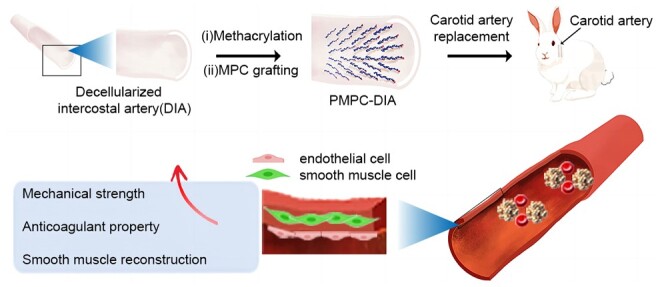

## Introduction

The prevalence and mortality rates of coronary heart disease (CHD) remain high, presenting one of the most significant challenges in the global public health issues [1, 2]. The World Health Organization reports that CHD is responsible for approximately nine million deaths annually, accounting for nearly 16% of global mortality. Coronary artery bypass grafting (CABG) utilizes the patient’s own vessels to create bypasses around narrowed or blocked arteries, thereby restoring blood supply to the ischemic myocardium [3]. However, such treatment suffers several critical limitations and certain drawbacks. Primarily, CABG often relies on the patient’s own saphenous veins, internal mammary arteries, or radial arteries as the autografts. Inherent limitations are obvious, such as the availability and quality of these autologous vessels, coupled with the fact that not all patients have vessels healthy or suitable for artery bypass. Furthermore, harvesting vessels from other parts of the patient’s body often causes additional surgical trauma and risks, leading to further complications such as wound healing issues, infections, and pain, which adversely impacts overall recovery and quality of life [4]. Additionally, though autologous vascular grafting generally provides effective coronary blood flow restoration in the short term, the long-term patency rates vary markedly between different sources of the autografts. Vein grafts have a 10-year patency rate of about 60%, in contrast to approximately 90% for arterial grafts [4]. Therefore, to address this clinical imperative, the utilization of the arterial xenografts is of the most urgent concern. This situation has created substantial clinical demands for vascular xenografts, especially the artificial small-diameter arterial grafts. However, current small-diameter vascular grafts (SDVGs, <6 mm) have far failed the expectations to compromise both biological and mechanical properties of natural vessels. These grafts are prone to thrombosis, caused by abnormal blood flow. Due to the unsatisfactory long-term patency rates, no mature SDVG product has been available at bedside [5].

The decellularized tissue grafts often preserve most of the extracellular matrix components and original structures from the native tissues, while the antigenic cellular components are removed. The decellularized vascular grafts, for example, not only exhibit good biocompatibility, but also shown superior capabilities in facilitating endogenous cell adhesion, growth, and tissue remodeling [6]. Presently, there are two predominant approaches in constructing tissue-engineered SDVGs. The first approach involves combination of decellularized matrix hydrogels and other functional materials to form small-diameter conduits. This method offers good biocompatibility and hemocompatibility, albeit with suboptimal mechanical and suture properties [7]. The second approach entails the decellularization of fresh small arterial vessels followed by proper modifications, producing vessel grafts with good mechanical properties but compromised hemocompatibility, which have mostly been prone to thrombosis [8]. Additionally, the great saphenous veins have also been employed as the tissue sources for preparing decellularized vascular scaffolds. However, the significant mismatch in diameters often leads to intimal hyperplasia, when compared with the autologous small arteries. Furthermore, the veins only consist of intima and adventitia, which barely support smooth muscle regeneration [9, 10].

**Figure 1.**
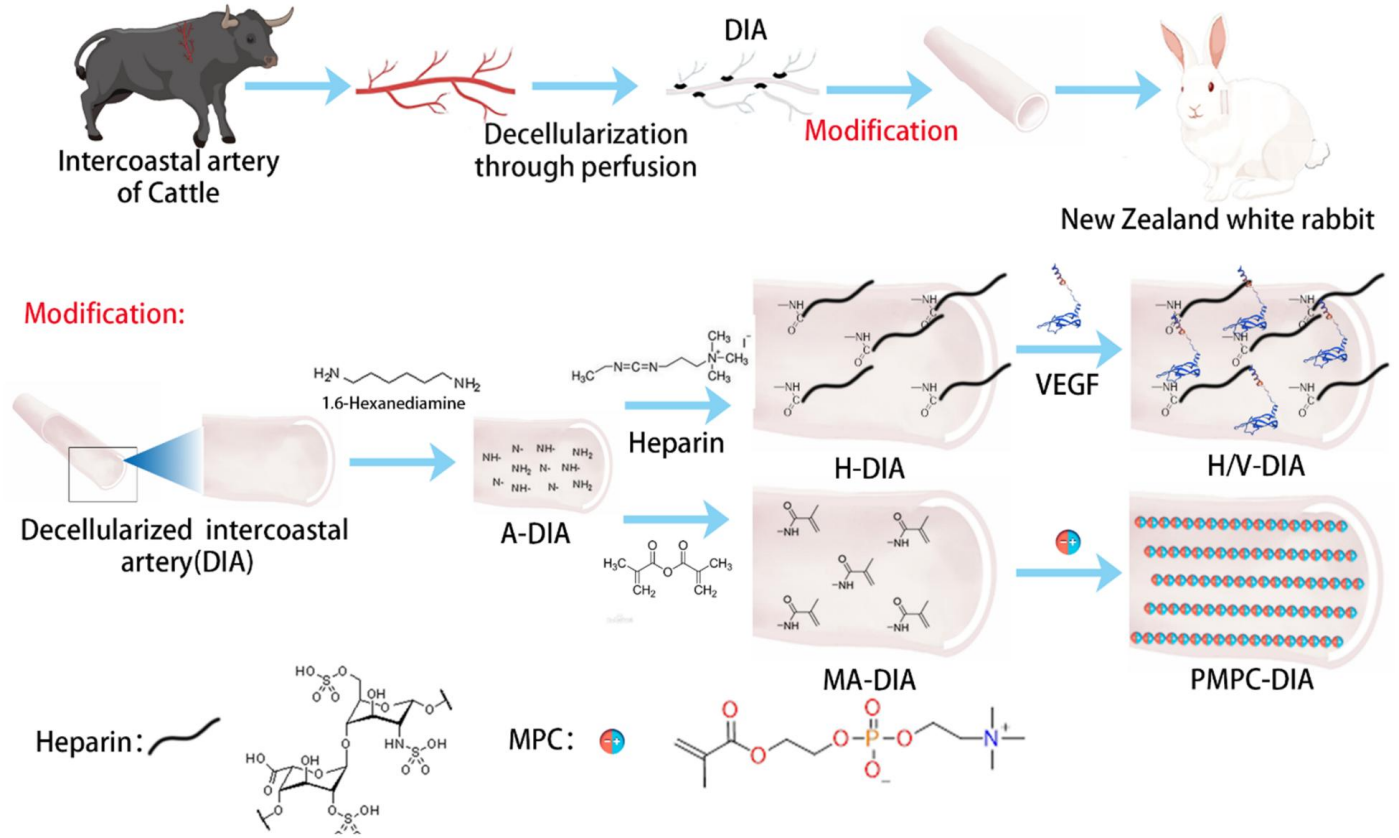
Schematic diagram showing the preparation of DIAs and two surface modification strategies post-decellularization. Some of the image materials were created by Figdraw.

In this study, certain significant advantages have been explored by using bovine-derived decellularized intercostal arteries (DIAs) as the small-diameter artery scaffolds, followed by one-pot reactions for DIA crosslinking and surface modifications prior to *in situ* implantation. These artificial artery grafts were found matching with the inner diameters of human coronary and other small-diameter arteries, which are available in lengths ranging from 15 to 30 cm. Furthermore, two different routes of inner surface modifications were investigated to introduce enhanced blood compatibility, mechanical properties and bioactivities toward artery vessel regeneration (Figure 1). Presumably, this study provides certain viable insights into fabrication of artificial small-diameter arteries, which showed that the resulting surface-modified DIA grafts can serve as promising candidates for clinical translation.

## Materials and methods

### Materials

The materials and reagents used in this study include phosphate buffered saline (PBS, 60-00-4, Sigma Aldrich, USA), penicillin-streptomycin (15140122, Life Technologies, USA), 2-[4-(2,4,4-trimethylpentan-2-yl) phenoxy] ethanol (R538531, Triton X-100, Sigma Aldrich, USA), sodium dodecyl sulfate (SDS) (V900859, SDS, Sigma Aldrich, USA), 4% paraformaldehyde (BL539A, Biosharp, China), heparin sodium (PHR8927, Sigma Aldrich, USA), vascular endothelial growth factor A (SEA143O, VEGFA, Cloud-clone, China), 1,6-hexanediamine (N801941, Macklin, China), 1-ethyl-3-(3-dimethylaminopropyl) carbodiimide (D4029, EDC, Samchun Pure Chemicals, Korea), 2-methacryloyloxyethyl phosphorylcholine (M84935, MPC, Macklin, China), methyl acrylate (M27301, Sigma Aldrich, USA), ammonium persulfate (A801035, APS, Macklin, China) and sodium bisulfite (S818005, SBS, Aladdin, China). For histological analysis, the reagents used for H&E staining, Masson’s trichrome staining, Alcian blue staining, Elastica van Gieson (EVG) staining, and Sirius red staining were all purchased from Servicebio, China.

### Harvest and decellularization of intercoastal artery

Intercostal arteries were extracted from fresh bovine ribeye steaks (obtained from local slaughterhouses) and then preserved in 100 U/ml penicillin and 100 g/l streptomycin in ice-cold PBS. The branches of the arteries were ligated, the connective tissues around the arteries were removed completely. A continuous circulated perfusion system was employed for decellularization of the dissected intercostal arteries. Each intercostal artery was cannulated using a 14-gauge needle and secured with suture knots, and then connected to a peristaltic pump to achieve a continuous flow of solution through the vessel (flow rate ∼50 ml/min). After removing the residual blood clots by saline perfusion, the intercostal arteries were decellularized by perfusing 1%(w/v) Triton X-100 solution and 1%(w/v) sodium dodecyl sulfate solution, respectively for 48 h each. The DIAs were then preserved in penicillin (100 U/ml) and streptomycin (100 g/l) containing PBS solution.

### Characterizations of DIAs

Residual DNA was quantified using a commercial DNA kit (G3330, Servicebio, China), and different histological analyses were performed to assess the degree of decellularization. Briefly, both native vessels and DIA samples were fixed in 4% paraformaldehyde for 24 h, dehydrated in graded alcohol (75%, 85%, 95% and 100%), and embedded in paraffin. According to the manufacturer’s instructions, paraffin samples were cut into 5 μm sections, deparaffinized with xylene, and then stained with hematoxylin and eosin (G1076, H&E, Servicebio, China), and DAPI (C1002, Beyotime Biotechnology, China), respectively. Masson’s trichrome staining (G1006, Servicebio, China) and Sirius red staining (G1078, Servicebio, China) were used to identify the distribution of collagen. Alcian blue staining (G1027, Servicebio, China) was employed to examine the glycosaminoglycans. Finally, EVG staining (G1042, Servicebio, China) was applied to characterize the elastic fibers. All the histological stained specimens were observed using an optical microscope (NIKON ECLIPSE E100, Japan).

### Preparation of DIA scaffolds modified by heparin and VEGF

To reduce the thrombogenicity of the decellularized scaffolds, the inner surfaces of the obtained DIA scaffolds were modified by heparin/VEGF and poly(2-methacryloyloxyethyl phosphorylcholine) (PMPC), respectively. For the heparin/VEGF surface modification, the DIA were first cut into 2-cm-length segments and then immersed in isopropanol solution containing 10% (w/v) 1,6-hexanediamine for 2 h [11]. Sodium heparin (40 mg) was added to 20 ml sodium citrate buffer (pH adjusted to 5.5) containing 40 mg EDC and agitated at 4°C for 24 h. The resulting DIA scaffolds with additional amines (A-DIA) were then immersed in heparin solution and incubated for 24 h at 4°C to allow heparin grafting [12]. After rinsing with distilled water, VEGF was introduced to the DIA scaffold by immersing in VEGF solution (100 ng/ml) in PBS at 4°C for 24 h [13]. The resulting heparin alone and heparin/VEGF modified DIA scaffolds were abbreviated as ‘H-DIA’ and ‘H/V-DIA’, respectively.

To quantify the release profiles of heparin and VEGF, the H-DIA and H/V-DIA scaffolds were immersed in PBS solution and the supernatant was collected after 5, 10, 15, 20, 25 and 30 days, respectively. Toluidine blue solution (T818873, Macklin, China) (0.04% w/v, in 0.01 M hydrochloric acid and 0.20% w/v NaCl) was added to the H-DIA supernatant and the samples were placed in 96-well plates to measure the absorbance at wavelength ∼530 nm. The relative amount of heparin was determined using a standard curve. The released VEGF in H/V-DIA scaffold was quantified by using the VEGFA ELISA Kit (SEA143Rb, Cloud-clone, China) according to the manufacturer’s protocol. The theoretical densities of heparin and VEGF was respectively calculated by the following formula,

$$\mathrm{Theoretical}\;\mathrm{density}=\frac{\mathrm{Total}\;\mathrm{amount}\;\mathrm{of}\;\mathrm{heparin}\;\mathrm{or}\;\mathrm{VEGF}}{\mathrm{Total}\;\mathrm{surface}\;\mathrm{area}\;\mathrm{of}\;\mathrm{the}\;\mathrm{graft}}$$

The total surface area was estimated by assuming the wall of the graft was thin enough,

$$\mathrm{Total}\;\mathrm{surface}\;\mathrm{area}=2\pi \times d_{i}\times l$$

where *d_i_* was the mean inner diameter of the graft, and *l* was the length of the graft.

### Fabrication of DIA scaffolds with PMPC surface grafting

The A-DIA scaffolds were connected to a perfusion apparatus and fully immersed in deionized water, then methacrylic anhydride (276685, Sigma-Aldrich, USA) was added dropwise to reach a final concentration ∼4% (v/v) at 4°C. The pH of the solution was adjusted to 7 using 5 M NaOH, and the methacrylation was maintained at room temperature for 24 h to obtain the methacrylated DIA (MA-DIA) [14]. The MA-DIA was then rinsed with deionized water to remove the residual reagents. After methacryloyl modification, MA-DIA was immersed in 3 M MPC solution, and then 50 mM APS and 50 mM SBS were added to the solution, then agitated at 37°C for 24 h [15]. The resulting PMPC-modified DIA (PMPC-DIA) scaffold was rinsed with deionized water and then stored in sterile PBS solution at 4°C until use.

### Free amine content

The quantification of free amino groups was implemented using ninhydrin assay. Both A-DIA and PMPC-DIA were cut into small pieces measuring roughly 0.5 × 0.5 cm, and each was placed into a centrifuge tube with 2 ml 1% ninhydrin solution (485-47-2, Alfa Aesar, USA) buffered with 0.1 M sodium citrate solution (pH = 5.0, HY-B1610J, MCE, USA). The samples were incubated at 95°C for 40 min, after which the absorbance of the supernatant was measured at 567 nm, corrected against a blank ninhydrin solution. A dilution series of glycine (56-40-6, Sigma-Aldrich, USA) were used as the standard. After treatment, each sample was rinsed three times with deionized water and dried at 45°C until a constant weight was reached, and the dry weight (W) of the sample was recorded. The conversion rate of amino groups was calculated using the following formula:

$$\mathrm{Conversion}\;\mathrm{of}\;\mathrm{amino}\;\mathrm{groups}\;\left(\%\right)=\left[1-\frac{\frac{{\mathrm{OD}}_{\mathrm{PMPC}}}{W_{\mathrm{PMPC}}}}{\frac{{\mathrm{OD}}_{A}}{W_{A}}}\right]\times 100\%$$

where the conversion of amino groups indicates the reduction or consumption of free amino groups, OD_PMPC_ represents the optical density of the PMPC-DIA scaffold, *W*_PMPC_ represents the weight of the PMPC-DIA scaffold, OD_A_ represents the optical density of the A-DIA sample and *W*_A_ represents the weight of the A-DIA sample.

### Scanning electron microscopy

After lyophilization, samples were carefully cut to expose the inner surface of the vessels and affixed to scanning electron microscopy (SEM) stubs by conductive adhesive tape. The samples were uniformly coated with a thin layer of platinum using a sputter coater (ISC150, SuPro, China) in argon atmosphere, and then observed using a scanning electron microscope (HITACHI SU8010, Japan) at 10 kV. Images were captured at 400× magnification, five to seven fields were randomly selected on each sample, and analyzed using ImageJ software (NIH, US).

### Surface elemental analysis

Prior to X-ray photoelectron spectroscopy (XPS) measurement, the samples were rinsed ethanol and blow-dried with nitrogen. The analysis was implemented using a Thermo SCIENTIFIC ESCALAB 250Xi XPS system (Thermo Scientific, USA). Survey scans were performed with pass energy ∼100 eV to identify the surface elements, followed by high-resolution scans at 20 eV for detailed chemical state analysis. Binding energies were calibrated to C1s peak at 284.8 eV, serving as a reference for hydrocarbon contamination. Data analysis, including peak deconvolution and quantification, was carried out using dedicated Avantage software (Thermo Scientific, USA).

Chemical constituents of the modified DIA scaffolds were examined through their specific wavelengths using Fourier transform infrared spectrometer (FT-IR, Nicolet iS10, Thermo Scientific, USA) with OMNIC version 7.3 software (Thermo Scientific, USA). The samples of 10 mm^2^ were analyzed across spectrum ranging from 4000 cm^−1–600^ cm^−1^, with measurements at intervals ∼4 cm^−1^.

### Mechanical characterizations

Uniaxial tensile strengths of the DIAs were measured using a universal tensile mechanical tester (WD-5A, ZYYD, China). The DIA scaffolds were clamped between the upper and lower grips with ∼10 mm distance, then stretched longitudinally at 10 mm/min until rupture. The samples maintained moist throughout the test. The stress-strain curves were measured using the software supplied with the machine, and the data was analyzed using Origin software (Originlab, USA).

The DIA scaffolds were cut into 0.5 cm in length for burst pressure measurements. One end of each specimen was connected to a pressure transducer (YK-100B, Yunyi, China) equipped with a three-way valve, while the other end was securely tied to ensure it was sealed. The vessel was then filled with saline solution. Through the three-way valve, more saline was gradually introduced into the vessel, thereby steadily increasing the internal pressure. The pressure at which the specimen burst was recorded as its burst strength by software SHILEK (Yunyi, China).

Statistical analysis of the stress-strain curves was performed using the data processing functions in Origin software, such as data smoothing and baseline correction, to optimize the quality of the data. Specific parts of the curves, such as Young’s modulus (the slope of the curve) and maximum tensile stress (vertical coordinate corresponding to the breaking point) and elongation at break (horizontal coordinate corresponding to the breaking point) were obtained using Origin graphing and curve fitting tools.

### Cytocompatibility

Fresh intercostal arteries, DIA, H/V-DIA and PMPC-DIA scaffolds (pre-sterilized by UV irradiation for 48 h) were cut into 1-cm-long specimens and then immersed in PBS for 48 h to obtain their extract solution, respectively. Human umbilical vein endothelial cells (HUVECs, Cat. 8000, Sciencell, USA) were seeded onto 96-well plates at ∼2000 cells/well in endothelial cell growth media (211-500, Sigma, USA). After culturing in a 37°C humidified incubator with 5% CO_2_ for two days, 10 μl scaffold extract of each group was added to the 100 μl culture medium, and further incubated for 24 h. The HUVEC-seeded tissue culture plates (TCPs) with the same cell density were used as control. CCK8 reagent (96992, Sigma-Aldrich, USA) was added into the media and incubated at 37°C for 3 h, and then the absorbance was measured at wavelength ∼450 nm by a Multiskan Mk3 microplate reader (5118160, Thermo Scientific, USA).

### Hemocompatibility

Rabbit blood was used to evaluate the hemolytic capabilities of the DIA scaffolds, and the whole blood (containing 10% v/v anticoagulant citrate dextrose) was diluted with PBS solution at 9:1 ratio [16]. Fresh intercostal arteries, DIA, H/V-DIA and PMPC-DIA scaffolds were cut into 10-mm-long grafts and placed in six-well plates, then 1 ml diluted blood mixture was added to each well. The pristine diluted blood mixture was used as the negative control. After incubation at 37°C for 1.5 h, the samples were centrifuged at 3000 rpm for 3 min, then the hemoglobin contents in the supernatant were measured at wavelength ∼540 nm with a Multiskan Mk3 microplate reader (5118160, Thermo Scientific, USA). The hemolysis rate of each sample was normalized by the positive control (blood incubated in deionized water).

For coagulation analysis, 100 μl whole blood was introduced to each 10-mm graft and maintained at 37°C for 30 min. Following specified intervals, the grafts were washed with distilled water to release trapped hemoglobin, which was then quantified using the same method as in the hemolysis test.

The platelet-rich plasma was isolated from whole blood by two consecutive centrifugation steps, first at 250× g for 5 min and then at 1800× g for 20 min. The platelet-rich plasma was then resuspended in PBS with the same concentration of whole blood, 100 μl platelet solution was applied to the luminal surface of each 10-mm sample and incubated at 37°C for 30 min. Subsequently, the scaffolds were transferred to fresh plates. The platelet dehydrogenase activity was quantified by the LDH activity assay kit (E-BC-K046-M, Elabscience, China) by following the manufacturer’s guidelines.

### Carotid artery replacement surgery in rabbits

All the experimental procedures used in this study were performed in compliance with the principles for the care and use of laboratory animals and was approved by the Institutional Animal Care and Use Committee (IACUC) of Sun Yat-sen University (No. SYSU-IACUC-2020-000509).

Five to six months old New Zealand white rabbits (*n* = 20) were randomly allocated into four groups, including DIA (2), H-DIA (6), H/V-DIA (6) and PMPC-DIA (6) groups, respectively. The rabbit carotid artery replacement surgery was established as previously described [17]. Briefly, the rabbits are general inhalational anesthetized by isoflurane (1–3%) delivered in oxygen at flow rate 1–2 l/min. Each rabbit was shaved and sterilized on the neck. An incision was made along the midline of the neck to expose the carotid artery. Then, the carotid artery was carefully isolated while protecting the surrounding nerves and tissues from injury. Once a sufficient length of carotid artery was isolated, the blood flow was temporarily blocked with vascular clips. After removal of a 2-cm-section of the carotid artery, the artificial vascular implants (DIA, H-DIA, H/V-DIA and PMPC-DIA) were sutured to the incision region of the original arteries by end-to-end anastomosis, respectively. The vascular clips were then released, while no obvious bleeding was noted at the vascular replacement sites. The healthy carotid artery on the non-operation side was performed as the control group. The incision was closed in a layer-by-layer manner. Appropriate postoperative management was performed, including the administration of antibiotics and analgesics, and the recovery of animals was monitored.

Ultrasound characterization of the carotid artery in each rabbit was carried out using Ultrasounder (Vetus E7 Pro, Mindray, China) with a high-frequency linear probe 30 days post-surgery. The rabbits were anesthetized and positioned supinely, with the neck area prepared for imaging. The probe, coated with ultrasound gel, was applied to visualize the artery, capturing images and measurements under standard settings optimized for vascular assessments. Finally, the rabbits were subject to euthanasia four weeks after surgery.

### Histological and immunofluorescence analysis

The implanted vascular grafts were collected and fixed in 4% formaldehyde, dehydrated in graded alcohol, embedded in paraffin and sectioned into 5-µm thick slices. The sections underwent antigen retrieval and endogenous peroxidase blocking process, then subjected to H&E staining, Masson trichrome staining, and Sirius red staining, respectively. For immunofluorescence staining, the tissue slides were blocked with 10% goat serum for 30 min and then incubated with primary antibodies including CD31 (ab213175, Rabbit, Abcam, US) and α-SMA (BM0002, Mouse, Boster, US) at 4°C overnight. The specimens were then rinsed with PBS and treated with secondary antibodies, including HRP-labeled goat anti-rabbit IgG (5110-0010, 488 nm, SeraCare, USA) and HRP-labeled goat anti-mouse IgG (5110-0011, 594 nm, SeraCare, USA), incubated for 50 min at room temperature. The nuclei were stained with DAPI for 5 min, and then rinsed with PBS solution. Images were captured under a fluorescence microscope (Axio Observer Z1, Zeiss, Germany). Quantification of fluorescence regions (pixels/mm^2^) was performed using ImageJ software.

### Statistical analysis

All tests were statistically evaluated using one-way Student’s t-test or two-way ANOVA using GraphPad Prism 8 software, with a minimum *P *<* *0.05. Unless otherwise stated, all relevant observations were repeated for least three times.

## Results

### Preparation and characterizations of DIA scaffolds

**Figure 2.**
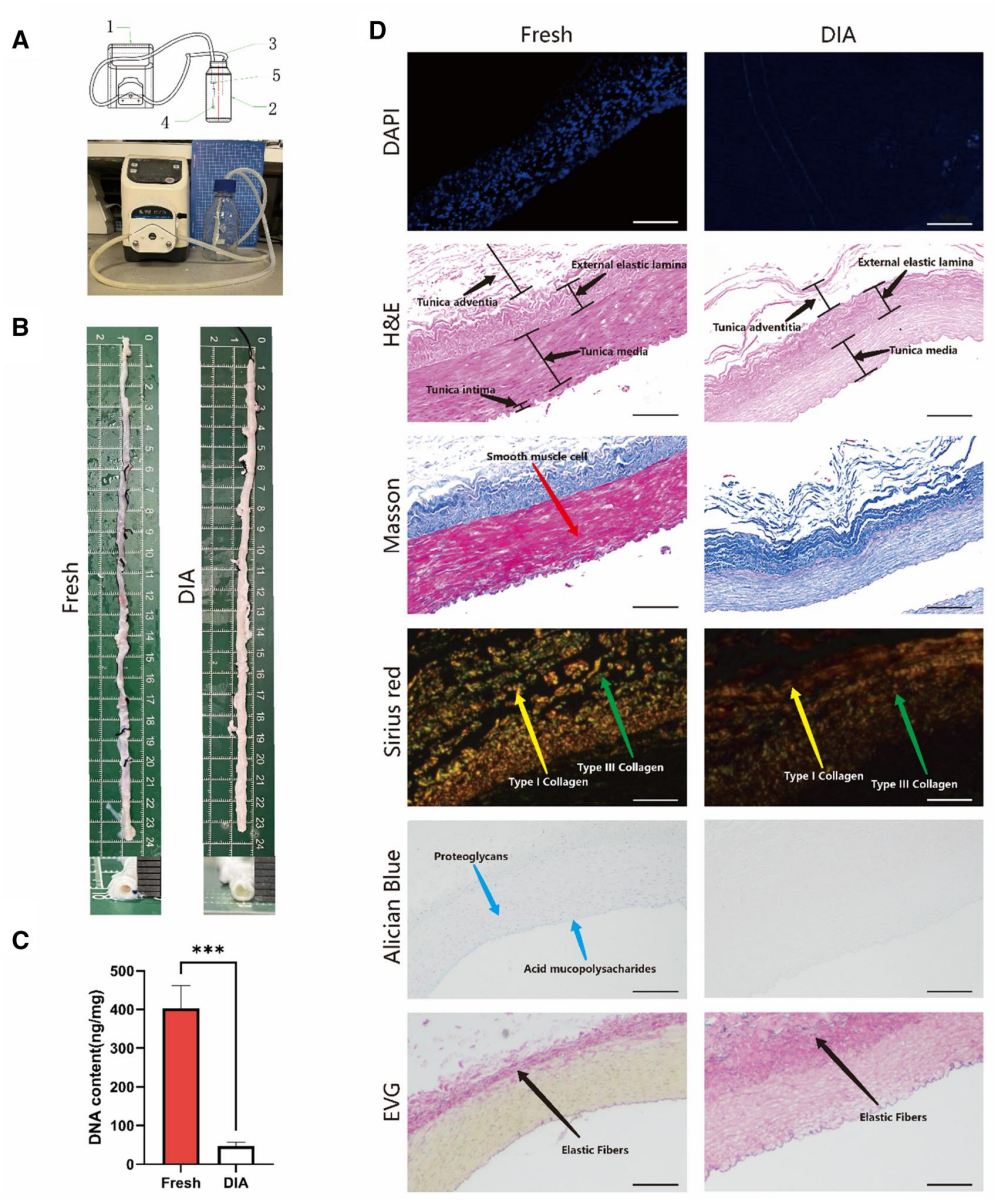
Preparation and characterizations of the DIA scaffolds. (**A**) Schematic illustration and photograph of the customized apparatus for decellularization (1: peristaltic pump, 2: blue cap bottle, 3: peristaltic pump tubes, 4: intercostal artery vessel, 5: needle). (**B**) Representative macroscopic photographs of both fresh intercostal artery and a DIA scaffold. (**C**) DNA contents quantified in both fresh intercostal arteries and DIA scaffolds. (**D**) Histological staining characterizations of the fresh intercostal artery and DIA scaffold, which include DAPI, H&E staining, Masson’s trichrome staining, Sirius red staining, Alcian blue staining, and EVG staining, scale bars = 250 μm. *n* > 3, ***P <* *0.001.

The intercostal arteries were decellularized using a customized perfusion system shown in Figure 2A. The DIA scaffold remained the gross shape of native intercostal artery from 15 to 30 cm in length, the cross-sectional images confirmed that the tubular structure was unobstructed and the inner diameter was ∼2 mm (Figure 2B). The residual DNA content decreased from 361.9 ± 126.5 ng/mg to 48.1 ± 10.6 ng/mg after decellularization (Figure 2C), while immunofluorescence staining confirmed that cell nuclei were completely removed. Both H&E and Masson trichrome staining revealed a three-layer laminated structure in the native intercostal artery including the tunica intima, tunica media, and tunica adventia [18], while the endothelial cells in the endothelium layer and smooth muscle cells in the media layer were obviously removed after decellularization (Figure 2D). Histological results showed that collagen I, collagen III, and acidic proteoglycans were retained with minor reduction after decellularization, characterized through Sirius red (using polarized light microscope) and Alcian blue staining. Meanwhile, results from EVG staining confirmed that the elastic fibers of the intercostal artery also preserved [19]. Collectively, it was demonstrated that both bioactive molecules and structural proteins of the extracellular matrix components were well-preserved in the DIA scaffolds.

### Characterizations of the surface modified DIA scaffolds

**Figure 3.**
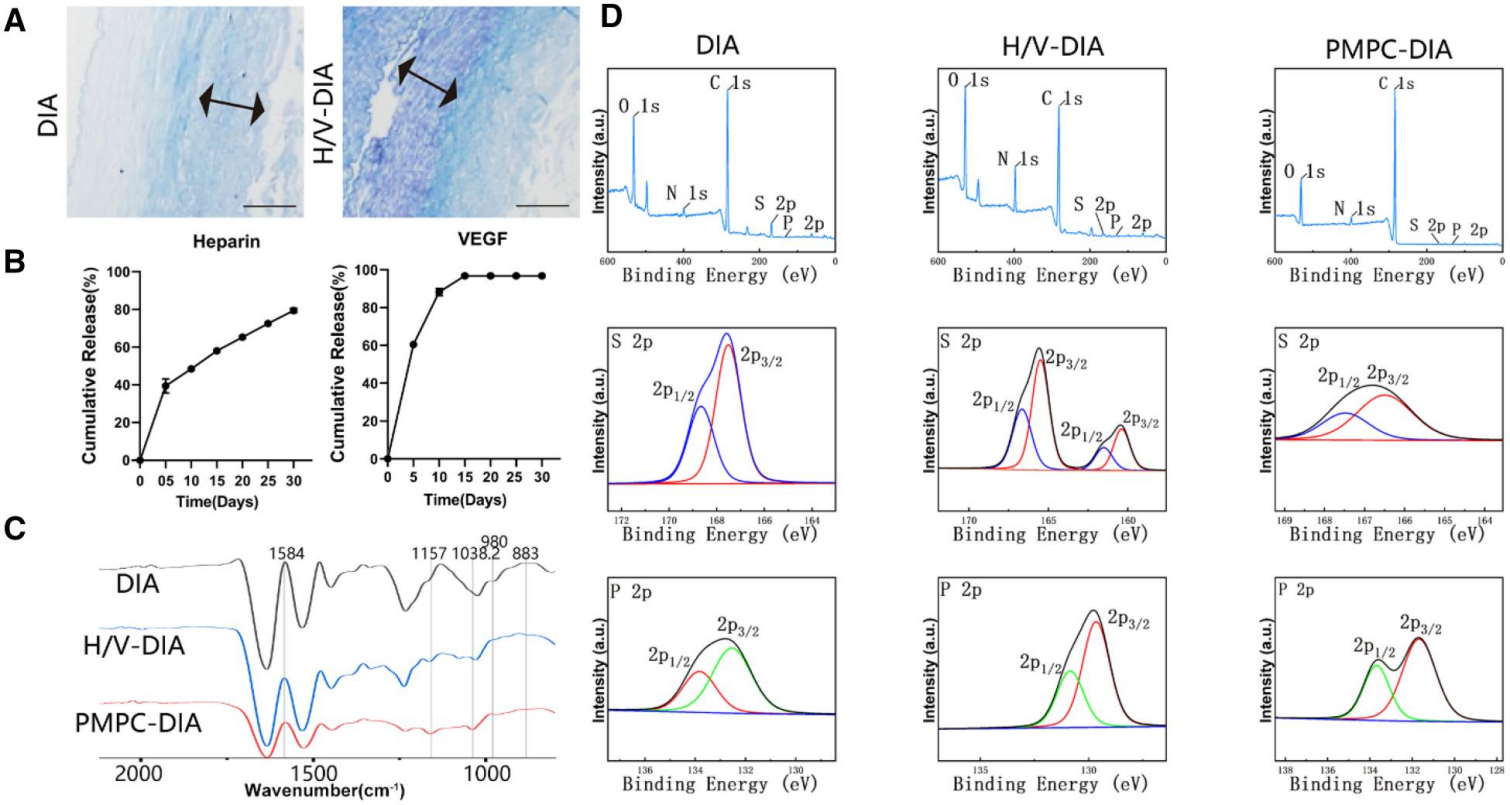
Characterizations of the inner-surface-modified DIA scaffolds. (**A**) Toluidine blue staining on both DIA and H/V-DIA scaffolds, scale bars = 500 μm. (**B**) Cumulative release profiles of heparin and VEGF from the H/V-DIA scaffolds. (**C**) FT-IR analysis of the DIA, H/V-DIA and PMPC-DIA scaffolds, respectively. (**D**) XPS spectra and elemental analysis of the DIA, H/V-DIA and PMPC-DIA scaffolds, respectively.

To improve the anticoagulation property of the decellularized artery vessels, both heparin/VEGF coating and zwitterionic PMPC grafting approaches were employed to modify the inner surface of the DIA scaffolds, respectively. First, it has been well-acknowledged that the sustained release of both heparin and VEGF is crucial to regulate biocompatibility and functions of the artificial vessels post-implantation [20]. Following heparin and VEGF modification, toluidine blue staining showed a significant heparin binding to the inner surface of the H/V-DIV (Figure 3A). The *in vitro* release profiles revealed sustained release of heparin throughout 30 days, while VEGF was completely released within 2 weeks (Figure 3B). The densities of heparin and VEGF (per unit inner surface) were calculated to be 7.99 ± 1.19 IU/cm^2^ and 785.34 ± 112.16 ng/cm^2^, respectively. Notably, VEGF release was faster than heparin, probably owing to the non-covalent binding of VEGF to heparin. It was noticed from XPS analysis that although the sulfur elemental content (exists mainly in heparin) decreased in the H/V-DIA scaffolds, the two distinct sulfur peaks of H/V-DIA indicated the presence of the sulfur element with another different valence state at 159–162 eV, compared to the characteristic peak at 166–170 eV of the DIA scaffolds, confirming heparin binding on the surface (Figure 3D).

Grafting amphiphilic polyzwitterion PMPC from the inner surface of the DIA scaffolds was primarily evident by the appearance of the absorption peak of the phosphate group (P = O) at 1157 cm^−1^ in the PMPC-DIA scaffolds compared to the DIA alone, based on the IR spectra (Figure 3C) [21]. The identification of two distinct absorption peaks at 130–132 eV and 133–135 eV of P2p in the PMPC-DIA scaffolds further confirmed the success grafting of PMPC brushes (Figure 3D). The conversion rate of free amine groups was approximately 90% (Figure S1). Furthermore, both XPS characterizations and EDS mapping of the phosphorus (P) element distribution indicated that the PMPC brushes were only grafted on the luminal surfaces of the PMPC-DIA scaffolds (Figures S2 and S3). These analytical results were found consistent with the expected elemental composition changes in the H/V-DIA and PMPC-DIA scaffolds, respectively, preliminary indicated the success of surface modifications on the artificial DIA grafts.

### Surface morphology and mechanical properties

**Figure 4.**
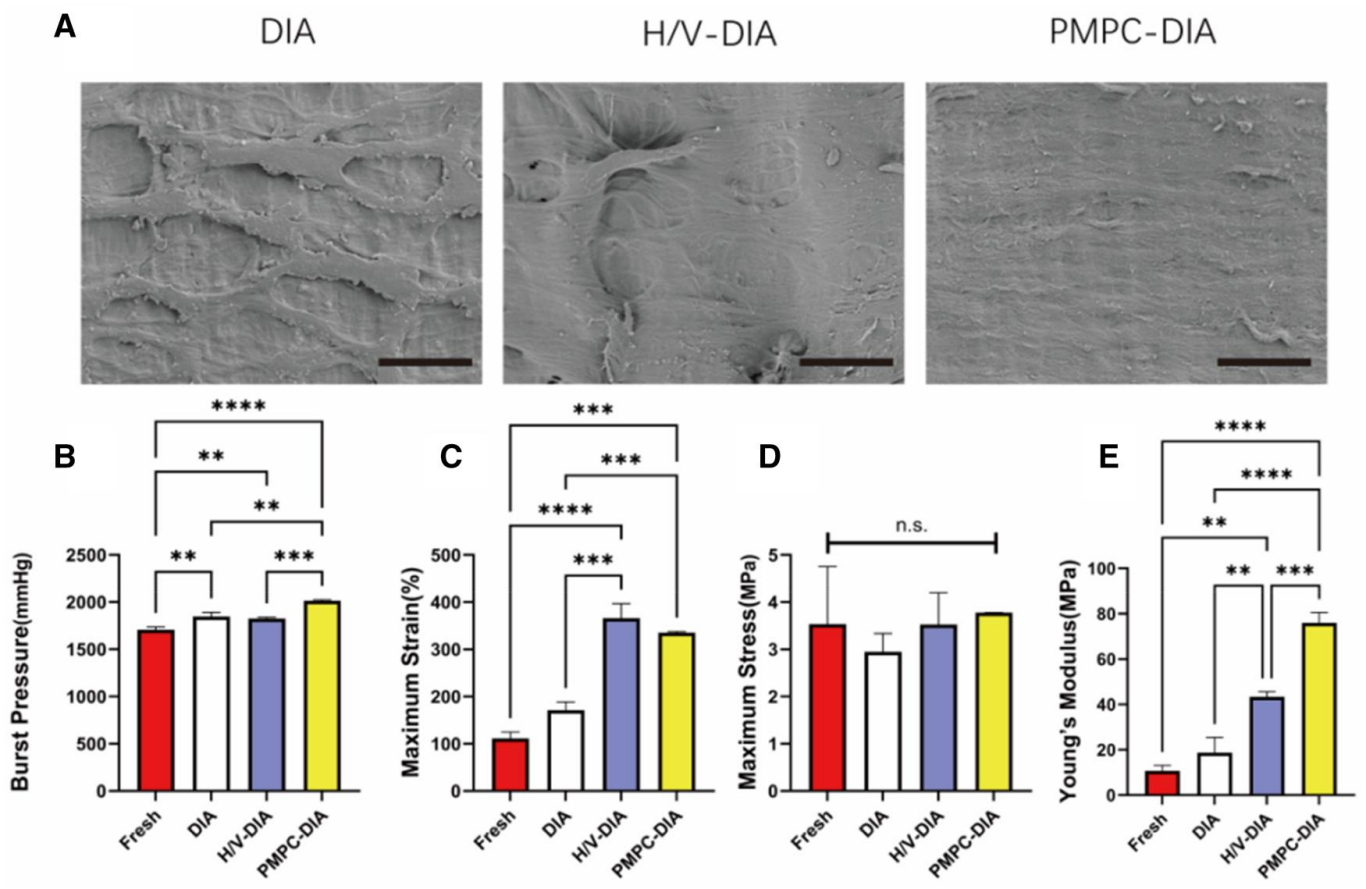
Microscopic observation and mechanical characterizations of the DIA scaffolds. (**A**) Representative SEM micrographs of the inner wall surfaces of the DIA, H/V-DIA and PMPC-DIA scaffolds, respectively, scale bars = 25 μm. (**B**) Bursting pressures of the fresh intercostal artery, DIA and modified DIA scaffolds. (**C**) Maximum strain, (**D**) maximum stress and (**E**) Young’s moduli of the fresh intercostal artery and different DIA grafts, characterized by longitudinal tensile tests. *n* > 3, **P <* *0.01, ***P <* *0.001, ****P <* *0.0001, and n.s. represents no significant difference between the compared groups.

After decellularization, it was noted that the inner walls of the DIA scaffolds presented a relatively rough surface with obvious fiber-like structures, characterized by SEM (Figure 4A). Comparatively, it was noticed that both inner surfaces of the modified DIA scaffolds became much smoother than that of the pristine DIA scaffolds. Respectively, the surface roughness of H/V-DIA scaffold was much smaller after heparin modification and VEGF loading. While the PMPC-DIA scaffold possessed the smoothest inner surface with nanofibers uniformly distributed, which may significantly contribute to reduction of platelet deposition and enhancement of anticoagulation property.

The mechanical properties of the decellularized vessels are critical for the patency of transplanted vascular grafts. Mechanical characterizations first showed that the burst pressure of fresh bovine intercostal artery was 1711 ± 23 mmHg, which was close to many other previously reported blood vessels derived from different vascular tissues (Figure 4B) [22]. After decellularization and consecutive surface modifications, the burst pressures of these modified DIA scaffolds increased, among which the PMPC-DIA scaffold reached the highest burst pressure by up to 1997 ± 36 mmHg. Uniaxial tensile strength testing showed that the maximum strain of fresh intercostal arteries and unmodified DIA scaffolds were 115.4 ± 8.2% and 154.1 ± 45.4%, respectively. Interestingly, the maximum strains of the H/V-DIA and PMPC-DIA scaffolds significantly increased to 360.5 ± 39.5% and 329.5 ± 12.2%, respectively, indicating a much better tensile resistance (Figure 4C). However, the maximum stresses of these scaffolds were close to each other among all four groups (Figure 4D). Due to the crosslinking effect during PMPC-grafting modification, the Young’s modulus of the PMPC-DIA scaffold was found significantly greater than those of the DIA alone and H/V-DIA scaffolds (Figure 4E). These results indicated that the surface-modified H/V-DIA and PMPC-DIA scaffolds exhibited good burst pressures and tensile strength, which were considered beneficial to graft patency.

### Cytotoxicity and hemocompatibility

**Figure 5.**
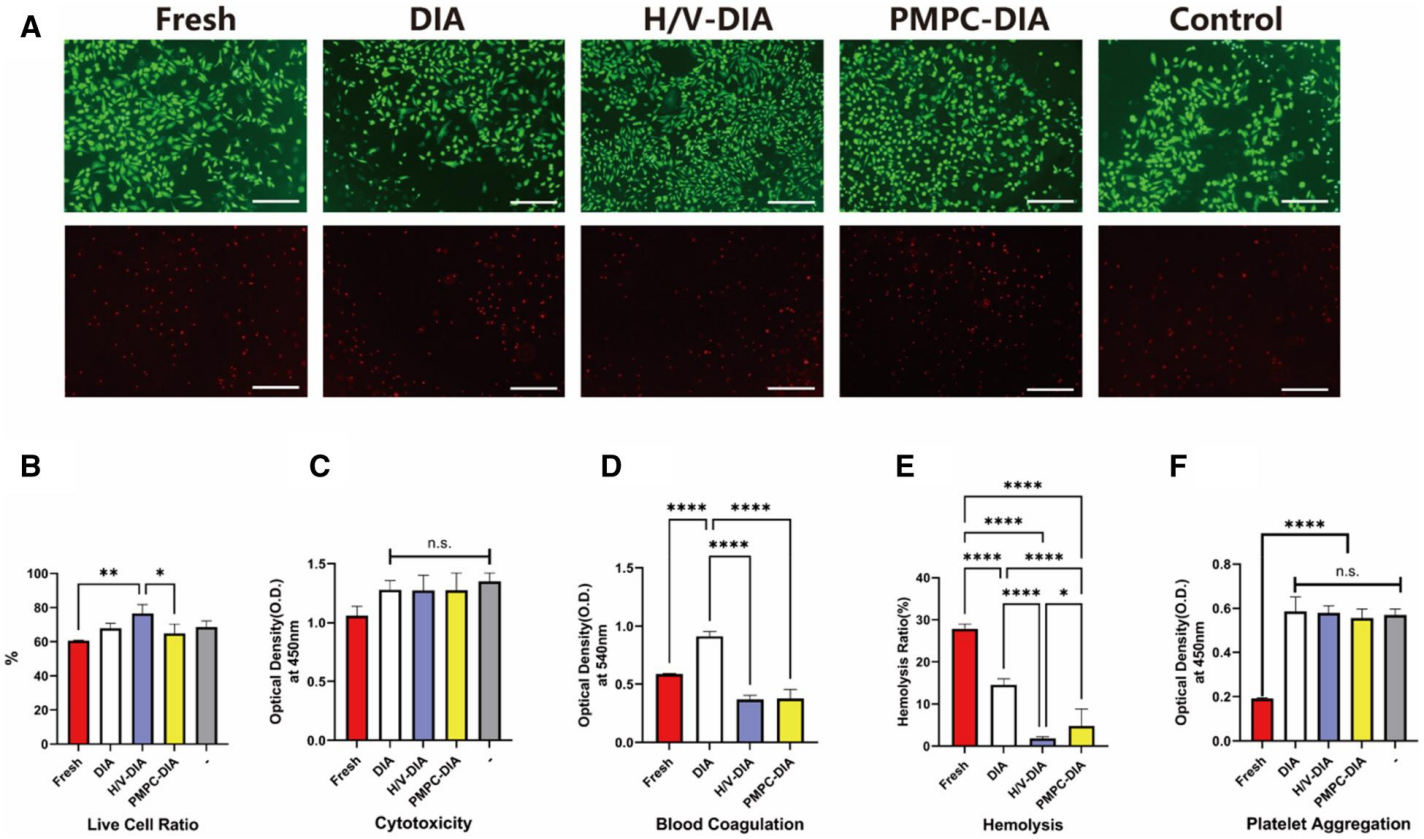
Biocompatibility and hemocompatibility of the fresh intercostal artery, DIA, H/V-DIA and PMPC-DIA scaffolds, respectively. (**A**) Live/dead staining after the HUVECs cultured for three days, with and without culture medium supplemented with extract solution of each graft, correspondingly, scale bars = 500 μm. (**B**) Quantification of the HUVECs viability, characterized by live/dead assay. (**C**) CCK-8 assay of the HUVECs cultured for three days, with and without medium supplemented with extract solution of each graft, correspondingly. ‘control’ and ‘–’ denote the same negative control group in which the HUVECs were cultured using normal medium. (**D**) Blood coagulation, (**E**) hemolysis and (**F**) platelet aggregation analysis of the different scaffolds. ‘–‘ group represents the negative control. *n* > 3, *P <* *0.05, **P <* *0.01, ****P <* *0.0001, and n.s. represents no significant difference between the compared groups.

To evaluate the biocompatibility of the modified DIA scaffold, the extract solutions of fresh intercostal artery, DIA, H/V-DIA and PMPC-DIA scaffolds were respectively collected and added to their corresponding media for culturing HUVECs. After 24 h of incubation, the HUVEC viability in the DIA, H/V-DIA and PMPC-DIA extract-containing groups were comparable to that in the control group (HUVECs cultured in TCPs alone), examined by Live/Dead staining (Figure 5A and B). It was noticed that the percentage of living cells in the H/V-DIA group was slightly higher than those of the other groups, owing to the presence and sustained release of heparin and VEGF in the medium, which benefited the growth of HUVECs. On the other side, results from CCK8 assay indicated that the DIA alone and surface-modified DIA scaffolds showed high bioactivities (Figure 5C), indicating relatively good biocompatibility of these artificial grafts.

Blood compatibility assessments were also performed by incubating fresh intercostal artery, DIA, H/V-DIA and PMPC-DIA scaffolds with whole blood and platelet-rich plasma, respectively. It was noticed that the DIA scaffold exhibited relatively weaker anticoagulant properties and poorer hemolysis resistance with neither surface modifications, indicating a need for further surface treatment to enhance hemocompatibility (Figure 5D and E). The clotting effect was significantly reduced within both H/V-DIA and PMPC-DIA scaffolds, which showed no significant difference compared to the negative control, implicating good anticoagulation properties. In the meantime, there was no significant difference in platelet agglutination, observed between the DIA alone, H/V-DIA, PMPC-DIA and the negative control group (Figure 5F). These might be attributed to the removal of the platelet-aggregation substances during decellularization.

### Implantation and vascular patency

**Figure 6.**
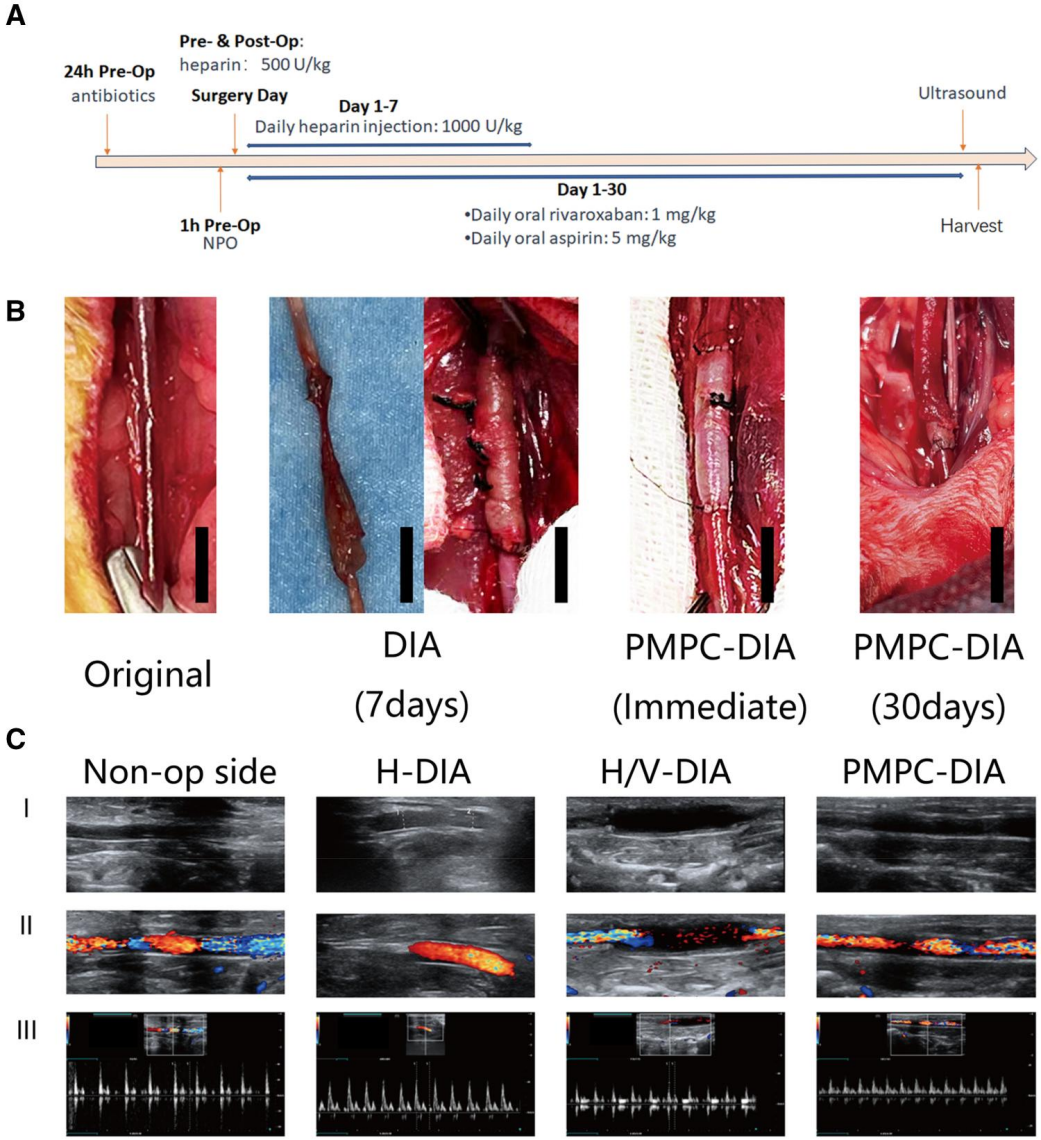
*In vivo* implantation of the modified DIA grafts using carotid artery replacement surgery in rabbits. (**A**) The timeline of the surgical procedure and sample harvest. (**B**) Representative photographs of the original carotid artery, as well as the implanted DIA and PMPC-DIA scaffolds post-surgery, respectively, scale bars = 1 cm. (**C**) Representative ultrasound images of the implanted H-DIA, H/V-DIA and PMPC-DIA scaffolds in rabbits dissected carotid arteries (I. B-mode ultrasound, II. Doppler ultrasound, III. ultrasound spectrum), the arteries of non-operated side were used as control (denoted as ‘Non-op side’).

To assess the impacts of decellularization and subsequent surface modifications on vascular patency, all DIA, H-DIA (heparin-modified DIA scaffold without VEGF integration), H/V-DIA and PMPC-DIA scaffolds were implanted into rabbits through carotid artery replacement surgery, respectively. The surgical management and experimental schedule are shown in the diagram (Figure 6A). Macroscopic images revealed that the original vessels maintained a normal appearance and color (Figure 6B). All the modified DIA grafts were filled with blood, the color and morphology of the vessels returned to a state close to that of the original vessels 30 days post-implantation. Unfortunately, all implanted DIA grafts without pre-modification failed the implantation assessment, due to non-visible blood flow and obvious stenosis within one week, which was found consistent with the poor anticoagulation properties of the DIA alone scaffold characterized *in vitro*.

Ultrasound characterizations intimately verified the patency of the implanted vascular grafts 30 days after surgery. Through plain ultrasound, Doppler ultrasound and spectral analysis (Figure 6C I, II and III, respectively), immediate restoration of blood flow was observed in the grafted modified DIA scaffolds post-surgery, which persisted for more than 30 days. It was evident by the restored blood flow velocity on doppler ultrasound and stable waveforms in spectral analysis. Statistical analyses confirmed that both peak systolic (PS) and end diastolic (ED) values of all the implanted H-DIA, H/V-DIA and PMPC-DIA grafts were close to each other (no significant difference was evident, Figure S4), indicating relatively good patency. These findings not only demonstrated that the well-performed physical patency of the implanted scaffolds, but also suggested functional restoration of the vascular endothelium, which is crucial for ensuring long-term success of the grafted vessels.

### Vascular remodeling in the modified DIA grafts

**Figure 7.**
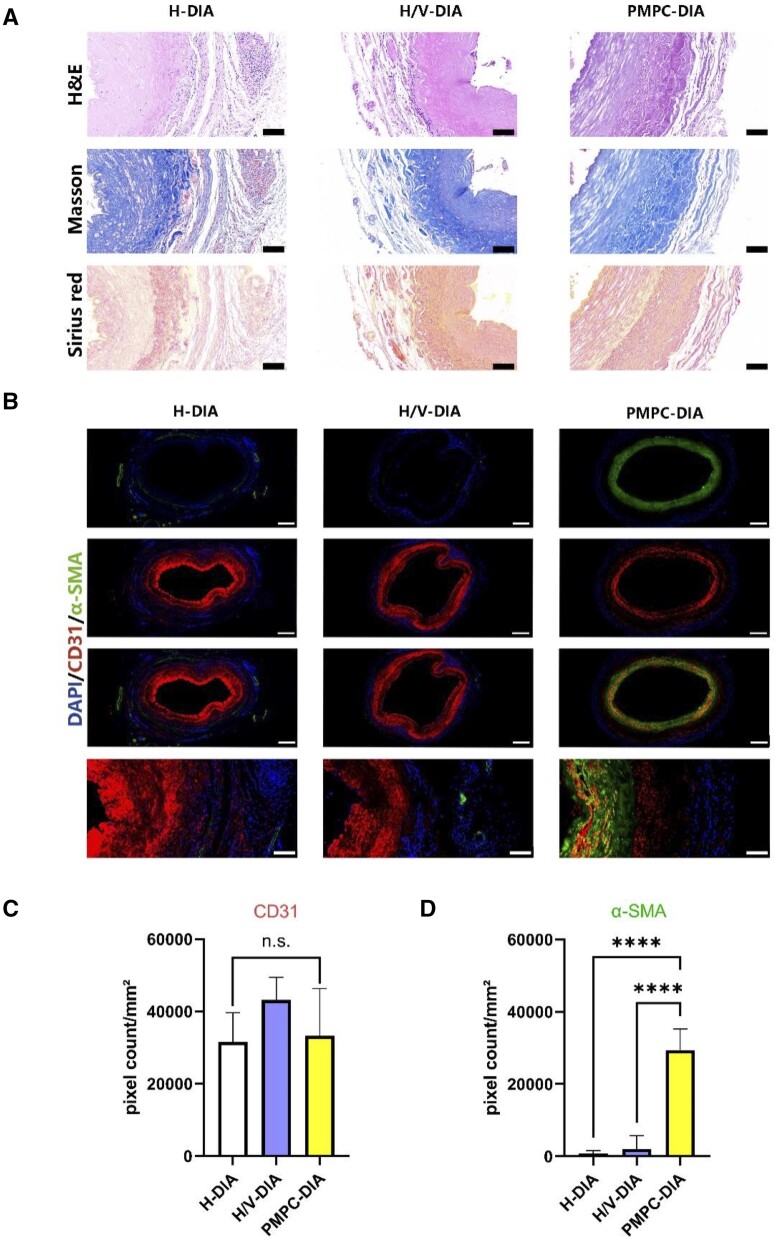
Histological and immunofluorescence analysis on the implanted vascular grafts 30 days post-surgery. (**A**) Representative cross-sectional micrographs showing the histological characterizations of the modified DIA scaffolds, H&E staining, Masson’s trichrome staining, and Sirius red staining, scale bars = 100 μm. (**B**) Immunofluorescence staining using CD31 (red), α-SMA (green) and DAPI (blue), scale bars = 500 μm for the upper three rows and 100 μm for the last row. Quantifications of the fluorescence area of (**C**) CD31 and (**D**) α-SMA based on the fluorescence micrographs representatively shown in (B). *n* > 3, ****P <* *0.0001, and n.s. represents no significant difference between the compared groups.

The structural integrity of the implanted vascular grafts was assessed using H&E, Masson’s trichrome, and Sirius red staining 30 days post-implantation. It was noted that intact arterial walls were clearly observed in all groups (Figure 7A). However, blurry distinction was noticed between the endothelium and mesothelium layers, as well as the presence of many inflammatory cells was evident in the H-DIA group. Results from Sirius red staining further demonstrated the presence of arterial structures, in which more defined endothelial layers were found in the H/V-DIA group, with endothelial cells appearing flattened and orderly distributed. Both results from Masson’s trichrome and Sirius red staining implicated that the presence and release of VEGF in the H/V-DIA group led to denser collagen fibers compared to that of the H-DIA group. Furthermore, compared to both heparin-integrated groups, the collagen fibers were found more orderly arranged in the PMPC-DIA group, and a layer of smooth-muscle-like fibers were also identified.

Furthermore, reendothelialization and regeneration of vascular smooth muscle after implantation of the modified DIA grafts were examined by immunofluorescence staining against biomarkers CD31 and α-SMA, respectively (Figure 7B). The results first confirmed that intact endothelial cell layers were evident in H-DIA and H/V-DIA after implantation, implicating the progress of reendothelialization. Although no significant difference was shown, statistical analysis on the fluorescence area suggested that the combination of heparin and VEGF in the H/V-DIA group led to enhanced endothelial recovery (Figure 7C). Meanwhile, the cross-sectional fluorescence micrographs showed that the vascular implants in the PMPC-DIA group exhibited notable smooth muscle reconstruction, evidenced by a continuous smooth muscle layer, unlike the sporadic presence of α-SMA signal in the other groups (Figure 7B and D). Interestingly, it was noticed that the extent of reendothelialization in the PMPC-DIA group was only comparable with that of the H-DIA group, but slightly lower than that of the H/V-DIA group (Figure 7C). It was conjectured that not only the integrated VEGF promoted reendothelialization for the H/V-DIA group, but the antifouling PMPC grafted luminal surface was also inhibitory to endothelial cell adhesion after implantation of the PMPC-DIA grafts. As the grafted vessels were remodeled gradually, the PMPC grafted inner layer might be delaminated by large blood pressure, reendothelialization was then triggered on the regenerated smooth muscle layers in the PMPC-DIA group (Figure S5).

## Discussion

Decellularized tissue-derived vessels have drawn great attention for CABG in recent years. Compared to the artificial grafts consisting of synthetic materials, the decellularized arteries retain the complex structures and extracellular matrix components in the native vessels, offering good biocompatibility and minimal immunogenicity [23, 24]. The biochemical cues preserved in decellularized artery also provide a suitable environment for re-cellularization and functionalization, which may reduce the risk of thrombosis and enhance the functionality of the scaffolds [25]. However, although some decellularized vessels with large-diameters have been used clinically for vascular transplantation, the application of decellularized arteries with small-diameters has been limited by several major drawbacks, basically a propensity for occlusion due to thrombosis, poor reendothelialization, inferior mechanical properties and reduced durability that may compromise long-term graft patency. Additionally, the process of decellularization is often more complex for smaller vessels, which can adversely affect the structural integrity of the vascular grafts. In this study, bovine-derived intercostal artery was chosen due to its widespread availability, appropriate caliber and sufficient lengths. After decellularization, it was observed that cellular components were essentially removed (Figure 2). Extracellular matrix components, such as collagen, elastic fibers, and glycosaminoglycans, were mostly preserved, providing a suitable platform for endothelialization that eventually contributes to inhibition of thrombosis and intimal hyperplasia. It was also noticed that the DIAs possessed slightly greater diameters compared to those of the fresh intercostal arteries (Figure 2B), which was possibly attributed to both surfactant-induced tissue hydration and relieving spasms after removal of the smooth muscle cells [26–28]. However, when implanted into rabbits, the DIA scaffold alone was found occluded in less than one week. It has been reported that small-diameter vessels were prone to occlusion mainly due to their small lumens, which often lead to changes in hemodynamics and increase the risk of thrombosis or intimal hyperplasia [29, 30]. Therefore, the inner walls of the DIA grafts require proper modification to improve the hemodynamics and vascular patency.

In recent years, several modification methods have been employed to reduce fibrin deposition, prevent thrombosis and promote reendothelialization. To increase the long-term patency, hydrophilic polymer brushes such as polyethylene glycol (PEG) and zwitterionic polymers (e.g. PMPC) were grafted onto the vessels to improve their antifouling capability and reduce thrombosis [31, 32]. On the other hand, bioactive molecules, such as gelatin, dopamine, or RGD (arginine-glycine-aspartic acid) sequences were introduced to the inner surface of the vessels to increase endothelial cell adhesion and migration [33–36]. Modification by heparin and VEGF has been a commonly used strategy, since heparin is often applied to enhance thromboresistance [33], and VEGF contributes actively to reendothelialization [37]. The modification with zwitterionic PMPC has also been demonstrated to impart various effects on materials. Formation of polyzwitterionic brushes on material surfaces significantly improves lubricity, which contributes significantly to improve hemodynamics through reducing friction and wear [38, 39]. Moreover, grafted PMPC brushes increased hydrophilicity and permeability, which are important for enhancing compatibility and tissue regeneration [40–42]. In this study, PMPC was grafted from the inner surface of the DIA scaffolds, and compared with the commonly used heparin/VEGF method. The sustained release profiles of heparin and VEGF, and the characteristic peaks shown in XPS and FT-IR spectra implicated the success of heparin/VEGF and PMPC modifications, respectively (Figure 3). After PMPC modification, SEM morphologies revealed that the surface became smoother compared to the DIA alone and H/V-DIA scaffolds, which was expected to be beneficial in subsequent blood flow smoothness (Figure 4A). The results of cell experiments and hemolysis tests also demonstrated the enhanced biocompatibility and blood compatibility of both H/V-DIA and PMPC-DIA scaffolds (Figure 5).

Meanwhile, PMPC grafting significantly increased the burst pressure and mechanical strength of the DIA scaffolds (Figure 4B–E), which was mainly owing to the crosslinking effects between the free methacryloyl groups within the MA-DIA scaffolds via free-radical-mediated chain-growth polymerization. Adequate burst pressure and tensile strength are crucial for maintaining the patency of vascular grafts [43, 44]. Both mechanical characteristics ensured the grafts to withstand both normal and abnormally high blood pressures without rupturing. This is particularly important for patients with cardiac conditions or hypertension, who may experience significantly higher blood pressures. High tensile strength is essential to maintain the structural integrity and openness of the grafts when subjected to blood flow impacts and body movements. Low tensile strength leads to easy deformation/elongation under physiological stress and changes in hemodynamics, which potentially results in complications, such as slowed blood flow or aneurysm formation. Moreover, grafts with sufficient mechanical strength can resist long-term biomechanical loads, reducing the risks of material fatigue and irreversible deformation that might lead to vascular narrowing and impeded blood flow. Additionally, a structurally stable graft not only supports blood flow, but also facilitates endothelial cell coverage and vascular repair, enhancing the graft integration with the host tissues, maintaining its long-term functionality and patency.

Compared to the obvious occlusion of the DIA alone scaffold within only 1 week post-implantation, the patency of the modified DIA vessels was significant improved and maintained for at least 30 days (Figure 6). Histological staining revealed that the addition of VEGF resulted in better intimal structure reconstruction, compared to the DIA scaffolds modified by heparin only (H-DIA). The PMPC-DIA scaffolds showed a much thicker media with clearer structural arrangement, and associated endothelial regeneration was comparable to that of the H-DIA group (Figure 7A and C). Notably, implantation of the PMPC-DIA scaffolds resulted in better reconstruction of the smooth muscle cell layer, rather than the H/V-DIA scaffolds. The fluorescence area of α-SMA+ signal was significantly lower in the H/V-DIA and H-DIA groups, while contiguous layer of smooth muscle appeared in the implanted PMPC-DIA grafts (Figure 7B and D), despite that slightly delayed reendothelialization was also evident in the PMPC-DIA group. This phenomenon was most likely attributed to the enhanced mechanical properties of the PMPC-DIA scaffold after methacrylation and subsequent crosslinking. It has been reported that tuning the compressive moduli modestly regulates cytoskeletal assembly, with more pronounced F-actin bundling in cells spreading in stiffer matrices [45]. Phenotypic changes of human coronary artery smooth muscle cells (HCASMCs) appeared to be modulated by the Young’s modulus and surface charge of the test substrates, indicating a structure-function relationship that can be exploited for intricate control over vascular cell functions [45–49]. Our results demonstrated that the PMPC-DIA scaffolds exhibited great anticoagulation capability and facilitated reconstruction of smooth muscles, demonstrating its great promise as functional artificial substitutes of the vascular autografts in clinical CABG surgery.

## Conclusion

In this study, a novel artificial vascular graft derived from decellularized bovine intercostal artery was successfully prepared and modified by zwitterionic PMPC brushes grafted from the luminal surface. Compared to the traditional heparin/VEGF modification strategy, the DIA scaffold modified by PMPC exhibited smoother inner wall and enhanced mechanical strength. Although both the H/V-DIA and PMPC-DIA scaffolds promoted vascular patency and reendothelialization compared to the DIA alone *in vivo*, only the PMPC-DIA scaffolds supported better smooth muscle fiber reconstruction, which is crucial for vascular functional recovery. This study not only provides a new viable source of decellularized arterial vessels with small-diameter, but also highlights the potential of PMPC-DIA scaffolds for future clinical applications in cardiac bypass surgery.

## Supplementary Material

rbae098_Supplementary_Data
