# Supplementary material for: Polyzwitterion-grafted decellularized bovine intercostal arteries as new substitutes of small-diameter arteries for vascular regeneration
Source: Regen Biomater. 2024 Aug 22;11:rbae098. doi: 10.1093/rb/rbae098 (PMC11368410; doi:10.1093/rb/rbae098)
Supplement: rbae098_Supplementary_Data [file rbae098_supplementary_data.docx]

Supplementary Information

**Polyzwitterion-grafted decellularized bovine intercostal arteries as new substitutes of small-diameter arteries for vascular regeneration**

Yuan Xia^1,†^, Zilong Rao^2,†^, Simin Wu^2^, Jiayao Huang^3^, Haiyun Zhou^4^, Hanzhao Li^4^, Hui Zheng^4^, Daxin Guo^4^, Daping Quan^2^, Jing-Song Ou^1,5,6,*^, Ying Bai^2,*^, Yunqi Liu^4,*^

1 Division of Cardiac Surgery, Cardiovascular Diseases Institute, The First Affiliated Hospital, Sun Yat-sen University, Guangzhou 510080, China

2 Guangdong Engineering Technology Research Centre for Functional Biomaterials, Key Laboratory for Polymeric Composite & Functional Materials of Ministry of Education, School of Materials Science and Engineering, Sun Yat-sen University, Guangzhou 510006, China

3 Department of Medical Ultrasound, The First Affiliated Hospital, Sun Yat-sen University, Guangzhou 510080, China

4 Department of Cardiac Surgery, The First Affiliated Hospital, Guangzhou Medical University, Guangzhou 510160, China

5 National-Guangdong Joint Engineering Laboratory for Diagnosis and Treatment of Vascular Diseases, NHC key Laboratory of Assisted Circulation and Vascular Diseases (Sun Yat-sen University), Key Laboratory of Assisted Circulation and Vascular Diseases, Chinese Academy of Medical Sciences, Guangdong Engineering Technology Centre for Diagnosis and Treatment of Vascular Diseases, Guangzhou 510080, China

6 Guangdong Provincial Key Laboratory of Brain Function and Disease, Zhongshan School of Medicine, Sun Yat-sen University, Guangzhou 510080, China

* Correspondence addresses. Tel: +86-20-84114030. Email: 710892613@qq.com (Y.L.); baiy28@mail.sysu.edu.cn (Y.B.); oujs@mail.sysu.edu.cn (J.O.)

† These authors contributed equally to this work.


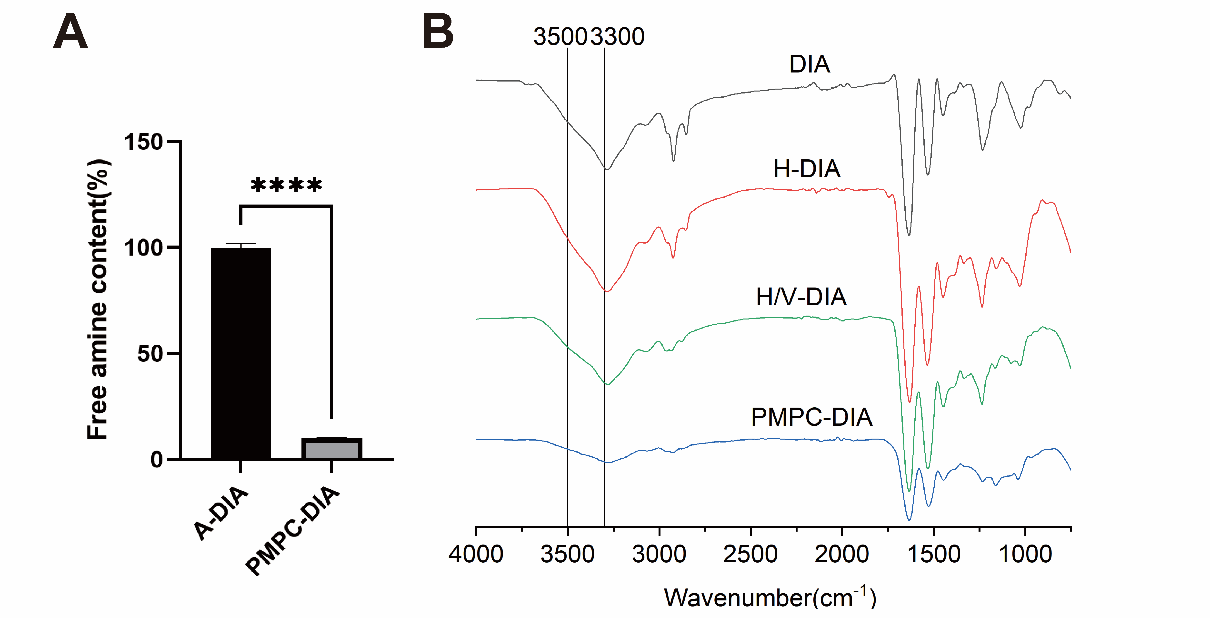


**Figure S1.** (A) Free amine content determined by ninhydrin assay (n = 6). The relative free amine content of the A-DIA scaffold was set as the 100% control. (B) Representative FT-IR spectra of the vascular grafts within wavelength ranging from 800 to 4000 cm^-1^. n > 3, ^****^*p* < 0.0001.


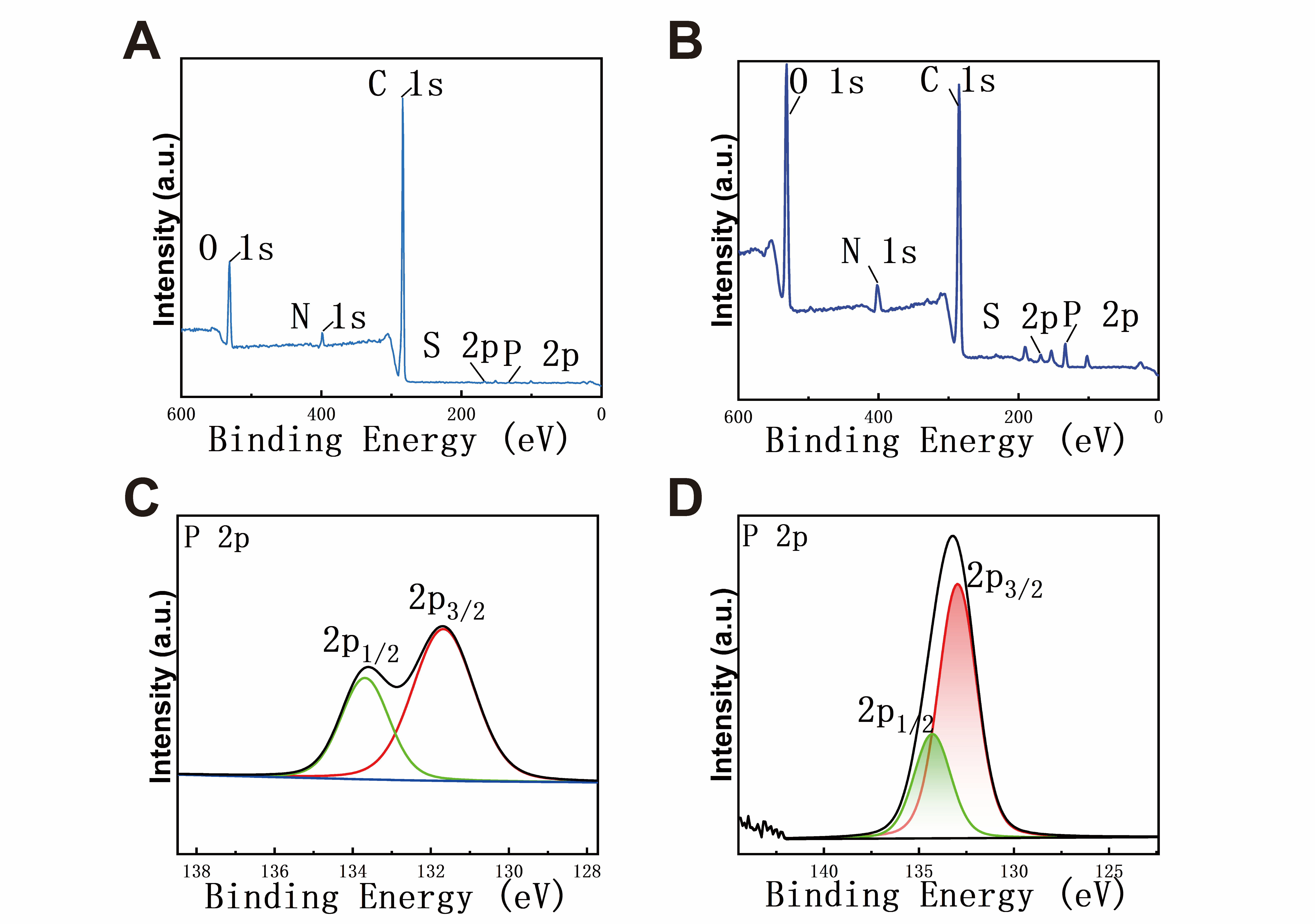


**Figure S2.** (A, C) Representative XPS characterization on the luminal surface of the PMPC-DIA scaffolds. (B, D) Representative XPS characterization on the cross-sectional regions of the PMPC-DIA scaffolds. Both (C) and (D) show the specific peaks of the phosphorus (P) elements.


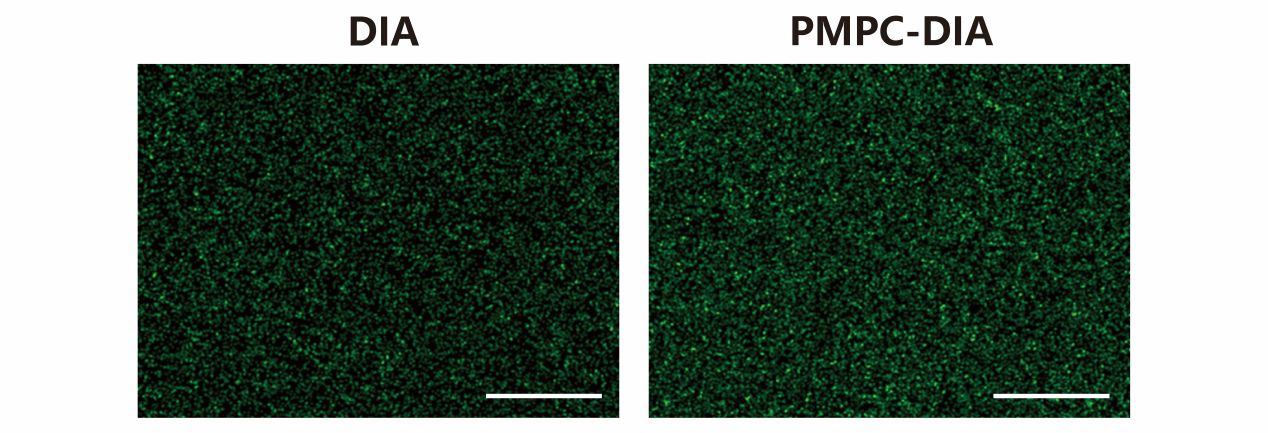


**Figure S3.** EDS mapping of phosphorus (P) element distribution on the luminal surfaces of the DIA and PMPC-DIA scaffolds, respectively. Scale bars = 25 μm.


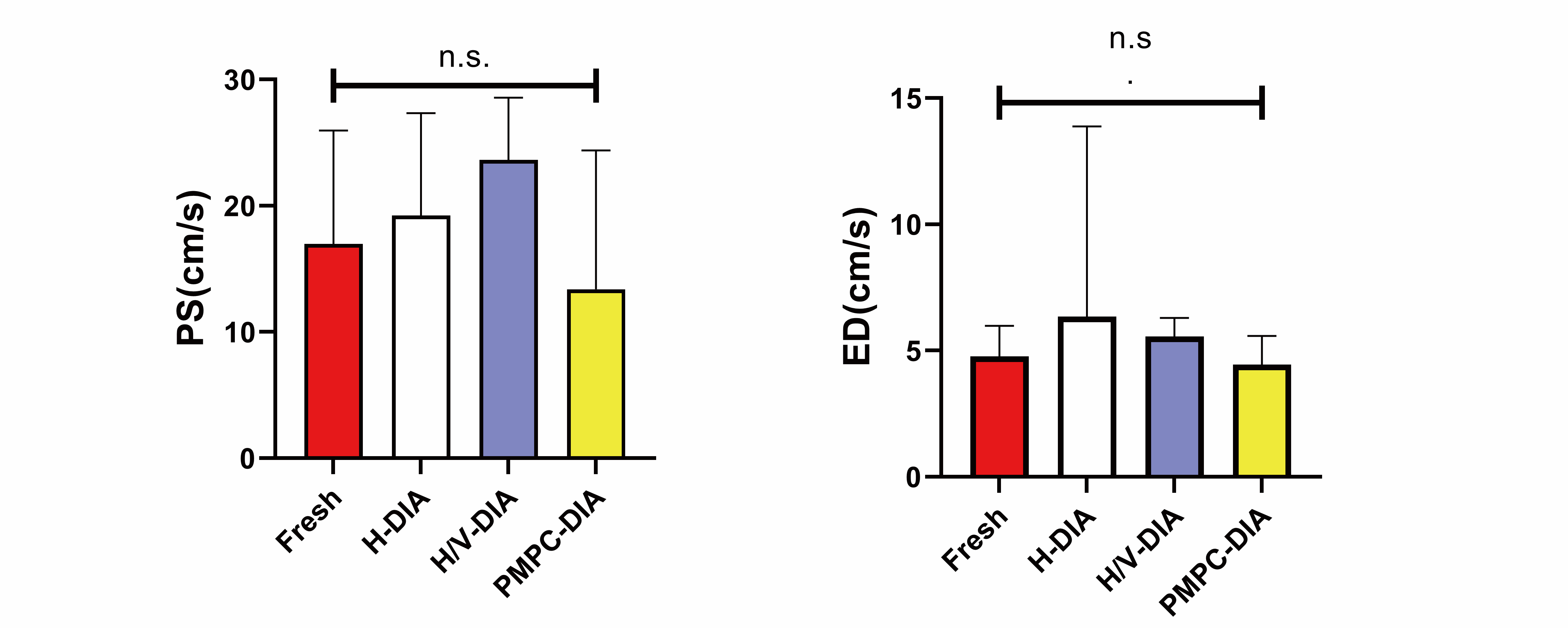


**Figure S4.** Peak systolic (PS) and end diastolic (ED) values of the implanted H-DIA, H/V-DIA, and PMPC-DIA grafts, respectively, recorded during ultrasound characterization, using fresh contralateral carotid artery as the control (Fresh). n = 6, and n.s. represents no significant difference.


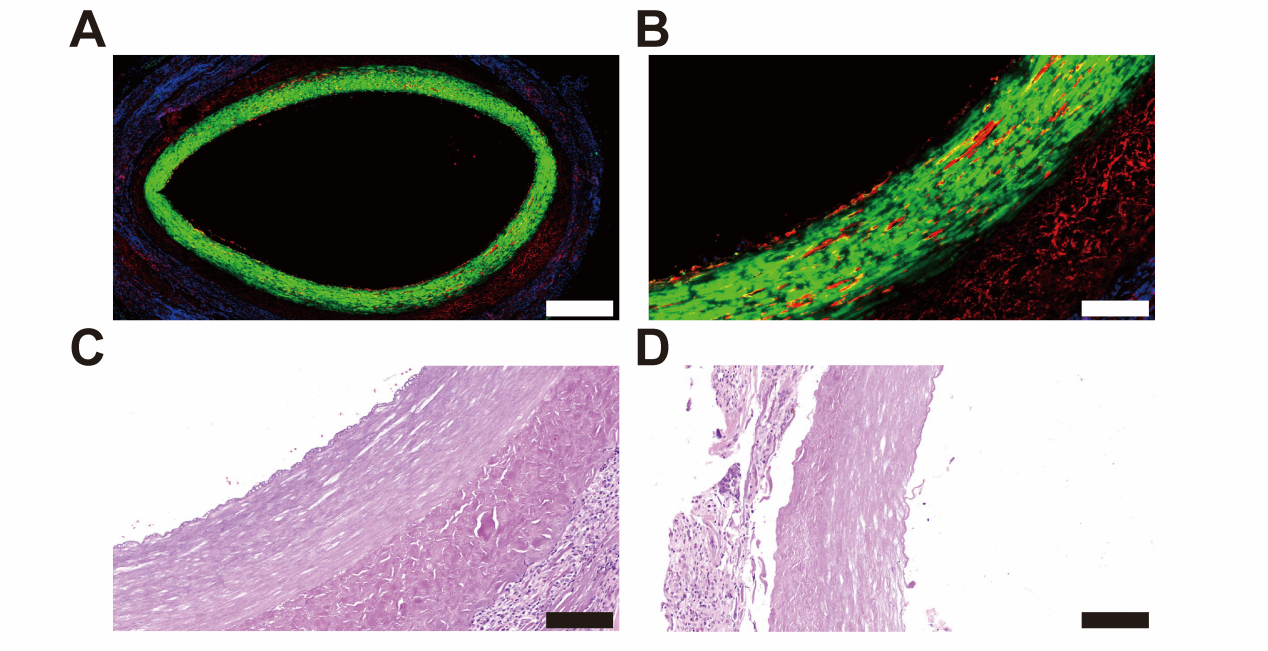


**Figure S5.** Representative immunofluorescence staining and histological staining micrographs showing endothelial regeneration effects in the PMPC-DIA group 30 days post-implantation. (A, B) Immunofluorescence staining on the cross-sectional regions of the implanted PMPC-DIA scaffolds using DAPI (blue) for nuclei, α-SMA (green) for smooth muscle cells, and CD31 (red) for endothelial cells, respectively. Scale bars = 500 μm in (A) and 100 μm in (B). (C, D) H&E staining results for histological analysis. Scale bars = 100 μm. (C) Representative PMPC-DIA graft with intact luminal and medium layers. (D) One of the PMPC-DIA grafts with slightly delaminated luminal layer.
